# Implementing a graduate medical education anti-racism workshop at an academic university in the Southern USA

**DOI:** 10.1080/10872981.2021.1981803

**Published:** 2021-11-23

**Authors:** Tina Simpson, Justin Evans, Alice Goepfert, Latesha Elopre

**Affiliations:** a Department of Pediatrics, University of Alabama at Birmingham, Birmingham, USA; b Department of Medicine USA, University of Alabama at Birmingham, Birmingham, USA; c Department of Obstetrics/Gynecology, University of Alabama at Birmingham, Birmingham, USA

**Keywords:** Anti-racism, graduate medical education, workshop, diversity, equity and inclusion

## Abstract

Coronavirus Disease 2019 (COVID-19) and the social justice movement in early 2020 awakened many Americans to the health disparities and health care inequities affecting Black communities. This heightened awareness has strengthened the call to address social determinants of health, like racism. Physicians can play an important role in dismantling racism through knowledge of implicit biases and understanding of historical trauma resulting in medical distrust as a crucial step to help advance the health of minority communities. The purpose of this project was to develop an anti-racism workshop for Graduate Medical Education. Two discussants led 1.5-hour interactive workshops. Content covered microagressions, colorblindness, tokenism, stereotypes, levels of racism, the impact of racism on health, and anti-racism concepts. Facilitated breakout sessions allowed participants to provide examples of witnessed racism and discuss application of anti-racism tools in those settings. Following the workshops, participants were asked to complete a 16-item survey to evaluate workshop effectiveness. Between July and August 2020, four workshops were delivered to 131 attendees. Fifty-nine completed post workshop surveys. Most respondents were White (75%), female (63%), and aged 31–40 (29%). Over half were faculty; 24% were residents, 8% fellows. The majority agreed they could apply knowledge to their work (95%) and found the workshop useful (95%). Over two-thirds reported being able to better identify disparities and better identify and communicate about racism. In open-ended questions, many participants requested an interactive longitudinal curriculum. Developing an antiracism workshop for an academic medical center located in the Deep South provided more insight into tangible next steps to foster an institutional culture centered on antiracism.

## Introduction

Coronavirus Disease 2019 (COVID-19) and the social justice movement in early 2020 *in response to the publicized murders of Black people* awakened many Americans to the health disparities affecting communities of color in the USA[1]. This heightened awareness has strengthened the call to include racism as a key social determinant of health that places people of color at risk for adverse health outcomes[2]. Racism has been proven to have direct (as well as indirect) impacts on health outcomes due to multiple psychophysiological pathways[3]. As such, leaders within Graduate Medical Education (GME) developed an anti-racism workshop for GME faculty, trainees, and staff to raise awareness on the impact of racism in healthcare and provide tools that individuals can utilize to dismantle racism.

## Materials and methods

### Content development

The virtual workshop was a 1.5-hour session, with 30-minute didactics separated by two facilitated 15-minute breakout sessions. The original didactic components covered: definitions and examples of health and healthcare disparities; an overview of the socioecological model, including racism; an explanation of race as a social construct [4]; manifestations of racism to include definitions of microagressions [5], colorblindness [6], tokenism [7], and stereotypes [8]; levels of racism (i.e., internalized, personally mediated, institutionalized) [9]; the impact of racism on health; and concepts of anti-racism [10]. Originally, the first breakout session centered on a discussion of health disparities and the second on using the concepts of antiracism to work toward dismantling racism within one’s own sphere of influence. This content was pilot tested with 13 workshop facilitators. Healthcare disparity content was reduced based on feedback. Additionally a pre-workshop assignment was added, the publicly available TedMed presentation by Dr. David R. Williams, *How Racism Makes Us Sick*. This video was approximately 17 minutes in duration to complete and no other pre-workshop exercises were included to avoid potential barriers to completion. To better demonstrate the levels of racism, a video of *The Gardener’s Tale*, an allegory by Dr. Camara Jones was added to the workshop. Breakout sessions were also restructured such that the first focused on a discussion of witnessed examples of racism after introduction of the concepts. The second breakout session allowed discussants to explore how anti-racism tools like ‘calling-in’ could be applied in settings when racism is witnessed[11]. Facilitators were asked to link individual examples from the first breakout session of experienced racism and ask the small group to elaborate on how they could apply concepts learned in those shared experiences. Small groups ended with a call to action as to how individual group members as well as community, institutions and policies could adopt antiracism concepts. Workshops closed with a ‘call to action’ resource provided by our university’s Office of Diversity, Equity and Inclusion, purposed for individuals to create their own action plan followed by resources on campus as well as literature on the topics presented.

### Workshop evaluation

A 16-item survey included demographics such as age, race, ethnicity, sex, gender identity, classification (i.e., faculty, staff, resident, fellow, student), and department. First, participants were asked, ‘Did you complete the pre-workshop assignment?’ for which they could answer yes or no. Using a five-point Likert scale, participants were asked: whether they would be able to apply information from the workshop to their work; if the workshop were a valuable use of their time; if they found the workshop to be useful; if they wanted to learn more about institutionalized racism; if they would recommend this type of workshop to others; if the section on health and healthcare disparities met their needs (i.e., meaning their subjective individual motivation for attending the workshop); if the section on racism met their needs; if the section on becoming an anti-racist met their needs. Additional survey items assessed whether participants thought differently about the impact of institutionalized racism on healthcare outcomes, and if so how. Participants were also asked to what extent they gained skills in identifying health disparities, healthcare disparities, and racism, as well as skills to communicate with colleagues and patients about racism.

## Results

Between July and August 2020, 207 GME faculty, staff and trainees registered for four workshops. The majority who registered were White (n = 139, 69%) and female (n = 126, 66%). Faculty represented 36% (n = 70) of those registered. (Table 1) Fifty-nine attendees completed post workshop surveys. Most respondents were White (n = 45, 75%), female (n = 37, 63%), and aged 31–40 (n = 29, 29%). Over half were faculty (n = 34, 58%); 13% (n = 13) were residents, and 10% (n = 6) medical students. The majority agreed they could apply knowledge to their work (n = 55, 94%), found the workshop useful (n = 55, 94%), and a valuable use of their time (n = 51, 87%). Nearly 97% (n = 56) of respondents would recommend the workshop to others. After the workshop, 75% (n = 44) thought differently about the healthcare impact of institutionalized racism. Over two-thirds (n = 48) reported being able to better identify and communicate about racism. In open-ended questions, respondents overwhelmingly felt the breakout sessions and overview of concepts around racism were the most helpful (Table 2). Additionally, many participants requested an interactive longitudinal curriculum with a series of lectures and discussions to build momentum around culture change.

**Table 1.** Demographics of anti-racism workshop and survey participants

Characteristics	Registered Participants (n = 207)^a^	Survey Participants (n = 59)
Age < 25 25–30 31–40 41–50 51–60 61–70	-	2 (3%) 13 (22%) 17 (29%) 10 (17%) 12 (20%) 5 (8%)
Race White Black Asian Other^b^ Declined to Answer	139 24 31 3 0	45 (75%) 5 (8%) 8 (13%) 0 2 (3%)
Ethnicity Non-Hispanic Hispanic Declined to Answer	188 9 0	54 (92%) 4 (7%) 1 (2%)
Sex assigned at birth Male Female Declined to Answer	71 126 0	21 (36%) 37 (63%) 1 (2%)
Gender Identity Male Female Other^c^ Declined to Answer	-	21 (36%) 37 (63%) 0 1 (2%)
Department Surgical^d^ Non-surgical^e^ Medical Student	-	10 (22%) 43 (73%) 6 (10%)
Position Faculty Resident Staff Medical Student	70 (36%) 52 (27%) 42 (21%) 32 (16%)	34 (58%) 13 (22%) 6 (10%) 6 (10%)

^a^131 out of 207 registered attended.

^b^Included: American Indian, Alaska Native, Native Hawaiian, Pacific Islander, Two or more races.

^c^Included: Transgender male, transgender female, non-binary, not listed.

^d^Surgical: OB/GYN, General Surgery, Urology, Orthopedic Surgery.

^e^Non-surgical: Anesthesiology, Dermatology, Emergency Medicine, Family Medicine, Internal Medicine, Neurology, Pathology, Pediatrics, Psychiatry, Radiology.

**Table 2.** Anti-racism workshop survey responses (n = 59)

Survey Question	Scale – Likert n (%)			
	Strongly Disagree or Disagree	Neutral		Agree or Strongly Agree
I will be able to apply information from the workshop to my work?	1(2)	2(4)		55(94)
The workshop was a valuable use of my time?	2(4)	5(9)		51(87)
Overall, I found the workshop to be useful?	1(2)	2(4)		55(94)
I want to learn more about institutionalized racism?	1(2)	3(5)		54(93)
I would recommend this type of workshop to others?	1(2)	1(2)		56(96)
The section on health and healthcare disparities met their needs?	5(9)	6(11)		47(80)
The section on racism met their needs?	1(2)	4(7)		53(91)
The section on becoming an anti-racist met my needs?	2(4)	8(14)		48(82)
	**No Change or Not Much**	**A little Bit**		**Quite a Bit or A Lot**
To what extent do you feel you have gained skills in: Identifying Health Disparities Identify healthcare Disparities Identifying Racism Communicating with Colleagues about Racism Communicating with Patients about Racism	10(18) 12(22) 7(13) 5(9) 9(16)	20(36) 16(29) 13(24) 19(35) 21(38)		25(46) 27(49) 35(63) 31(56) 25(46)
	**No**		**Yes**	
Do you think differently about the impact of institutionalized racism on healthcare outcomes as a result of this workshop	14 (25)		44 (75)	
	**Major Themes:**			
What you found most useful?	- Breakout sessions - Information on types of racism and anti-racism - Pre-video assignments			
What you found least useful?	- Recap on breakout sessions from facilitators - Technical difficulties related to sound			
What would you change?	- Series of talks and activities around racism and other marginalized groups - More diverse attendance (racial/ethnicity) - More brainstorming about actionable steps to change the institution - Shorter workshop duration or break workshop into two sessions			
What is one thing that you intend to do as a result of this workshop?	- Speaking up more and ‘calling in’ when witnessing racism - Learn more about racism			

## Discussion

By developing an antiracism workshop for an academic medical center located in the Deep South, insight was obtained on tangible next steps to foster an institutional culture centered on antiracism. While the Liaison Committee on Medical Education and the Accreditation Council for Graduate Medical Education mandate recruitment and retention efforts for trainees under-represented in medicine, there is less emphasis on training residents and fellows, as well as the faculty who educate them, to create a culture of belonging[12]. Additionally, antiracism interventions published in the literature more often focus on curriculum developed for medical education. There is a dearth in the literature on antiracism curricula for graduate medical education, where trainees reach a critical transition point where they are intimately engaging in patient care. We found through piloting and from survey feedback that most attendees were eager to learn about the different facets of racism and wanted the emphasis of the workshop focused on actions individuals, communities and institutions could take to dismantle racism. Additionally, attendees were eager to learn about resources available on campus that could be easily accessed. Importantly, we found that many White faculty had an immense desire to improve their understanding of systematic racism to foster an anti-racism culture within our institution, with a call for more curricula and discussions moving forward to prevent this workshop from being the end of formal didactics around this topic. As educators, we found it difficult to fit such a nuanced and intricate topic into a 1.5 hour workshop. While attendees called for more information on action, we felt the need to balance a need for knowledge acquisition first about this topic to change attitudes and potentially influence behaviors. A global pandemic and social justice movement have shed a brighter light on the role racism plays in health, necessitating more formalized curricula to address this in Graduate Medical Education for long-term gains to be seen in culture change and most importantly equitable patient care.

## References

[1] Poteat T, Millett G, Nelson LE, et Al. Understanding COVID-19 risks and vulnerabilities among black communities in America: the lethal force of syndemics. Ann Epidemiol. 2020;47:1–3. 10.1016/j.annepidem.2020.05.004 PMC7224650 32419765

[2] Paradies Y, Priest N, Ben J, et al. Racism as a determinant of health: a protocol for conducting a systematic review and meta-analysis. Syst Rev. 2013;2(1):85. 24059279 10.1186/2046-4053-2-85 PMC3850958

[3] Harrell CJP, Burford TI, Cage BN, et al. Multiple pathways linking racism to health outcomes. Du Bois Rev: Soc Sci Res Race. 2011;8(1):143. 10.1017/S1742058X11000178 PMC3328094 22518195

[4] Witzig R. The medicalization of race: scientific legitimization of a flawed social construct. Annals of Internal Medicine 125(8), 675-679. 10.7326/0003-4819-125-8-199610150-00008 8849153

[5] Sue DW, Alsaidi S, Awad MN, et al. Disarming racial microaggressions: microintervention strategies for targets, White allies, and bystanders. Am Psychologist. 2019;74(1):128. 10.1037/amp0000296 30652905

[6] Bonilla-Silva E, Dietrich D. The sweet enchantment of color-blind racism in Obamerica. ANNALS Am Acad Political Social Sci. 2011;634(1):190–4.

[7] Zimmer L. Tokenism and women in the workplace: the limits of gender-neutral theory. Social Problems. 1988;35(1):64–77.

[8] Gladwell M. Blink: the power of thinking without thinking. 2006. New York: Back Bay Books, Little, Brown.

[9] Jones CP. Levels of racism: a theoretic framework and a gardener’s tale. Am J Public Health. 2000;90(8):1212. 10936998 10.2105/ajph.90.8.1212 PMC1446334

[10] Kendi IX. How to be an antiracist. One world; 2019. New York: Random House.

[11] DiAngelo R, Sensoy Ö. Calling in: strategies for cultivating humility and critical thinking in antiracism education. Understand Dismantl Privil. 2014;4(2):190–203.

[12] Yousif H, Ayogu N, Bell T. The path forward—an antiracist approach to academic medicine. N Engl J Med. 2020;383(15):e91. 32961043 10.1056/NEJMpv2024535

